# Impact of SARS-CoV-2 on Male Reproductive Health: A Review of the Literature on Male Reproductive Involvement in COVID-19

**DOI:** 10.3389/fmed.2020.594364

**Published:** 2020-11-19

**Authors:** Weihang He, Xiaoqiang Liu, Liang Feng, Situ Xiong, Yulei Li, Luyao Chen, Yu Li, Gongxian Wang, Dongshui Li, Bin Fu

**Affiliations:** ^1^Reproductive Medicine Center, The First Affiliated Hospital of Nanchang University, Nanchang, China; ^2^Department of Urology, The First Affiliated Hospital of Nanchang University, Nanchang, China; ^3^Jiangxi Institute of Urology, Nanchang, China

**Keywords:** SARS-CoV-2, COVID-19, ACE2, tmprss2, gender differences, male fertility

## Abstract

Coronavirus Disease 2019 (COVID-19) has created a global pandemic. Global epidemiological results show that elderly men are susceptible to infection of COVID-19. The difference in the number of cases reported by gender increases progressively in favor of male subjects up to the age group ≥60–69 (66.6%) and ≥70–79 (66.1%). Through literature search and analysis, we also found that men are more susceptible to SARS-CoV-2 infection than women. In addition, men with COVID-19 have a higher mortality rate than women. Male represents 73% of deaths in China, 59% in South Korea, and 61.8% in the United States. Severe Acute Respiratory Syndrome Coronavirus 2 (SARS-CoV-2) is the pathogen of COVID-19, which is transmitted through respiratory droplets, direct and indirect contact. Genomic analysis has shown that SARS-CoV-2 is 79% identical to SARS-CoV, and both use angiotensin-converting enzyme 2 (ACE2) as the receptor for invading cells. In addition, Transmembrane serine protease 2 (TMPRSS2) can enhance ACE2-mediated virus entry. However, SARS-CoV-2 has a high affinity with human ACE2, and its consequences are more serious than other coronaviruses. ACE2 acts as a “gate” for viruses to invade cells and is closely related to the clinical manifestations of COVID-19. Studies have found that ACE2 and TMPRSS2 are expressed in the testis and male reproductive tract and are regulated by testosterone. Mature spermatozoon even has all the machinery required to bind SARS-CoV-2, and these considerations raise the possibility that spermatozoa could act as potential vectors of this highly infectious disease. This review summarizes the gender differences in the pathogenesis and clinical manifestations of COVID-19 and proposes the possible mechanism of orchitis caused by SARS-CoV-2 and the potential transmission route of the virus. In the context of the pandemic, these data will improve the understanding of the poor clinical outcomes in male patients with COVID-19 and the design of new strategies to prevent and treat SARS-CoV-2 infection.

## Introduction

In early December 2019, several cases of pneumonia of unknown etiology were reported in China (Wuhan City of Hubei Province). The pathogen was confirmed as a novel coronavirus (2019-nCoV) by the Chinese authorities on January 7, 2020 (1). At present, the International Research Committee on Taxonomy of Pathogens and Viruses officially named the pathogen as Severe Acute Respiratory Syndrome Coronavirus 2 (SARS-CoV-2). SARS-CoV2 has emerged as a novel β-coronavirus and is the causative agent of Coronavirus Disease 2019 (COVID-19) (2). Since the outbreak of COVID-19 in December 2019, the number of infected cases has increased exponentially, and it was declared a pandemic by the World Health Organization (WHO) on March 11, 2020. By August 1, 2020, SARS-CoV-2 has infected 17,786,110 people and caused 683,491 deaths (https://www.worldometers.info/coronavirus/). In the past two decades, coronaviruses have caused two serious pandemics, including SARS in 2002 and Middle East Respiratory Syndrome (MERS) in 2012 (3). Although they all belong to the β-coronavirus cluster, SARS-CoV-2 has caused more infections, deaths and economic disruptions. According to recent reports, COVID-19 is primarily transmitted through respiratory droplets and contact, and its main symptoms and signs include fever, dry cough, nasal congestion, fatigue, ageusia, lymphopenia, and dyspnea (1). The disease spectrum of COVID-19 ranges from mild and self-limiting respiratory tract illness to severe progressive pneumonia, multi-organ failure, and death (4). Notably, male individuals seem to be susceptible to SARS-CoV-2 infection, and their mortality rate is also high (5, 6). Coronavirus infection is known to cause orchitis in cats, and based on reports, orchitis is a complication of SARS (7, 8). Ebola virus and Zika virus can cause sexual transmission of the virus by contaminating semen (9, 10). However, similar findings have not been reported in SARS-CoV-2.

In this review, we comprehensively review COVID-19 with regard to its pathogenic mechanism, clinical manifestations, and gender differences from related literature. In addition, we explain the relationship between COVID-19 and the previous two coronavirus pandemics. The possible mechanism of orchitis caused by SARS-CoV-2 and the potential transmission route of the virus are explored, emphasizing the challenges faced by male reproductive health in this pandemic.

## Pathogenic Mechanism of SARS-CoV-2

### Virion Structure

**Figure 1:**
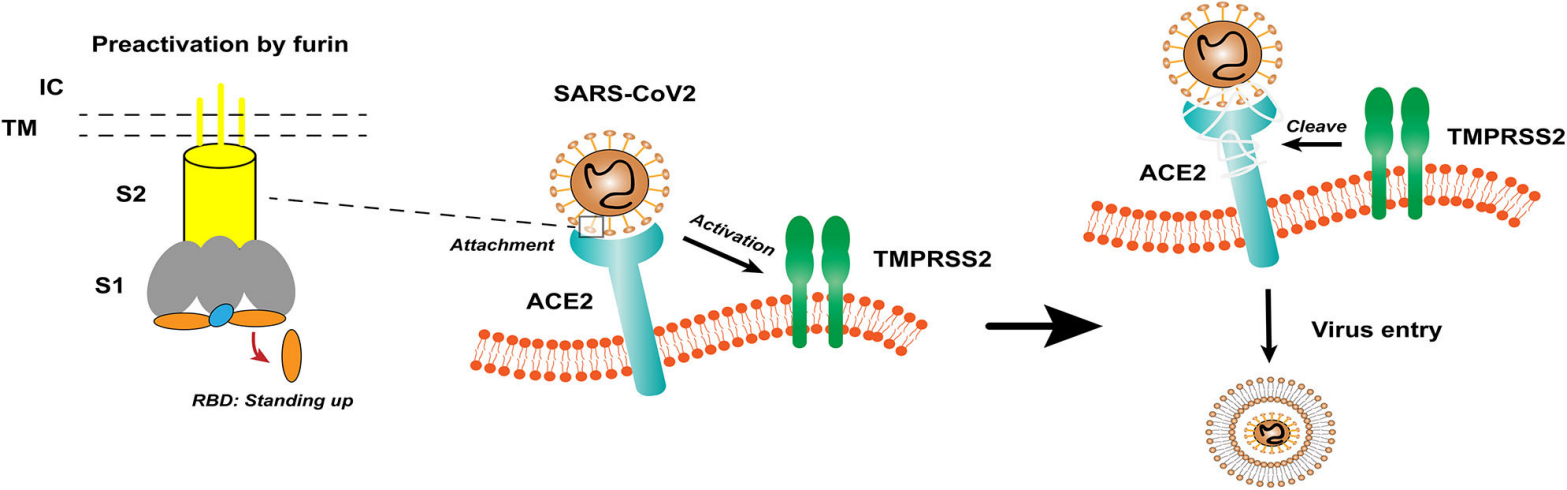
The structure of coronavirus spikes includes: S1, S2, TM, IC, RBD. The RBD hidden in the spikes for immune evasion. When recognizing the ACE2 receptor, the RBD stands up to bind the receptor. The furin pre-activation of the spike can enhance the ability of the virus to enter certain cells. At the cell membrane, SARS-CoV-2 recognizes the ACE2 receptor and recruits TMPRSS2. TMPRSS2 facilitates viral entry and spreads into the host cell by cleaving ACE2 and S protein. S1, receptor-binding subunit 1; S2, membrane fusion subunit 2; TM, transmembrane anchor; IC, intracellular tail; RBD, receptor-binding domain; ACE2, angiotensin-converting enzyme 2; SARS-CoV-2, Severe Acute Respiratory Syndrome Coronavirus 2; TMPRSS2, type II transmembrane serine protease.

Coronavirus (CoV) is an enveloped positive-sense RNA virus with special glycoprotein spikes around the viral envelope, showing a crown-like appearance under an electron microscope (2). With regard to genes, CoV is categorized into four important genera (*Alphacoronavirus, Betacoronavirus, Gammacoronavirus*, and *Deltacoronavirus*), which are the largest groups of viruses that cause respiratory and gastrointestinal infections. The α-CoV and β-CoV can infect mammals, whereas γ-CoV and δ-CoV predominantly infect birds (1). Structurally, coronavirus is composed of hemagglutinin esterase (only found in some β-CoVs), envelope, nucleocapsid, membrane, and spike (S) protein. S protein is an immense multipurpose viral transmembrane protein, and the entry of coronavirus into host cells is mediated by the interactions between S protein and its receptor (11). On mature viruses, the S protein exists as a trimer and contains two functional subunits, which mediate the binding to the host cell receptor (S1 subunit) and the fusion of the viral membrane and the cell membrane (S2 subunit) (12). Studies have shown that SARS-CoV has a receptor-binding domain (RBD) at the C-terminus of S1 (13). In addition, different coronaviruses use distinct domains within the S1 subunit to recognize various attachments to entry receptors (Figure 1) (12). A recent study has determined the crystal structure of SARS-CoV-2 RBD complexed with the receptor, revealing the subtle but important difference in receptor recognition between SARS-CoV-2 and SARS-CoV (14).

### Cellular Receptor for Coronavirus

Based on previous reports, the S protein of SARS-CoV binds to angiotensin-converting enzyme 2 (ACE2) as a host cell receptor. Given the genome sequence similarity between SARS-CoV and SARS-CoV-2, other studies have validated that SARS-CoV-2 also uses ACE2 as its receptor (12, 15, 16). On the other hand, proteases recruited by the virus to facilitate membrane fusion, especially TMPRSS2 (a type II transmembrane serine protease), which can cleave ACE2 and S protein, eliminate the structural constraint of S1 on S2, and releasing the internal membrane fusion peptide, thereby enhancing viral entry (Figure 1) (17). The full-length ACE2 consists of an N-terminal peptidase domain (PD) and a C-terminal Collectrin-like domain (18). The structure of the claw-like ACE2-PD alone and that complexed with the RBD of the S protein of SARS-CoV reveal the molecular basis of the interaction between the RBD of S protein and PD of ACE2 (19, 20).

ACE2 is a membrane exopeptidase, which is expressed in multiple organ systems, including the type I and type II alveolar epithelial cells, enterocytes of the small intestine, heart, kidneys, and testes (21, 22). The sequence of ACE2 is 41.8% identical to the domain of ACE (21). In addition, ACE2 plays a crucial role in the renin-angiotensin-aldosterone system (RAAS) (23). After angiotensinogen is produced in the liver, it is cleaved by renin to angiotensinogen (Ang) I, which is then converted to Ang II by ACE. Ang II induces bronchial smooth muscle contraction, pulmonary fibroblast proliferation, alveolar epithelial cell apoptosis, and pulmonary vascular permeability (24). By contrast, ACE2 acts as an angiotensin II-degrading enzyme to generate angiotensin (1-7), which has vasodilation, antihypertensive, and diuretic effects (25). Moreover, ACE2 participates in the absorption of neutral amino acids in the intestine (24). ACE2 has protective effects in multiple pathophysiological processes. The lack of protection of ACE2 leads to dysfunctional RAS and causes acute lung pathologies. Researchers found that infection of avian influenza H5N1, H7N9, and SARS-CoV results in a remarkably reduced ACE2 expression and subsequently elevates Ang II, which is associated with disease progression, severity, and lethality (26, 27). Furthermore, ACE2 can protect the lungs from acute lung injury. This protection is achieved by inactivating Ang II to negatively regulate RAS. The key positive role of ACE2 is not only in the respiratory system, but also in the modulation of heart function, kidney protection, and absorption of tryptophan in the epithelium of the small intestine (21, 22, 24). However, ACE2 plays an indispensable role in facilitating the cellular entry of SARS-CoV-2 and SARS-CoV. The duality of ACE2 has become the focus of recent research.

### Relationship With SARS-CoV and MERS Coronaviruses

In the past two decades, coronaviruses have caused two severe pandemics, including SARS in 2002 and MERS in 201 (3), both of which belong to the β-coronavirus cluster. The WHO has affirmed that SARS has caused 8,096 morbidities and 774 deaths in 2003, with a case fatality rate of 9.6%. By contrast, MERS has caused 2,494 cases and 858 deaths with a case fatality rate of 34.4% (1). SARS-CoV-2 is a novel β-coronavirus. Genomic analysis has shown that SARS-CoV-2 is ~79% identical to SARS-CoV (16), and it is the third zoonotic coronavirus disease and the third major medical crisis.

Despite the high case fatality rate of SARS-CoV and MERS-CoV, SARS-CoV-2 has caused more infections, deaths, and economic disruptions. By August 1, 2020, a total of 17,786,110 COVID-19 cases and 683,491 deaths were reported (https://www.worldometers.info/coronavirus/). Surface plasmon resonance technology was used to quantify the interaction kinetics of SARS-CoV-2-ACE2. The results show that the ectodomain of SARS-CoV-2 S protein binds to the PD of ACE2 with approximately 15 nM affinity, which is about 10- to 20-fold higher than that of SARS-CoV and ACE2 (28). A study has elucidated the structural and biochemical mechanisms of SARS-CoV-2 receptor recognition. The researchers have found that compared with SARS-CoV, the four residues responsible for coronavirus receptor binding in SARS-CoV-2 RBD have structural changes (residues 482–485: Gly-Val-Glu-Gly). The 3D structure of such residues shows a more compact configuration and form better contact with the N-terminal helix of ACE2 (14). In the loop conformation of the ACE2-binding ridge, the flexible glycyl residues of SARS-CoV-2 replace the rigid prolyl residues in SARS-CoV. The phenylalanine Phe486 of SARS-CoV-2 RBD is inserted into the hydrophobic pocket to provide an additional binding force (29). In addition, previous studies have identified two virus-binding hotspots (hotspot Lys31 and hotspot Lys353) and compared with SARS-CoV, both virus-binding hotspots are stabilized at the SARS-CoV-2–ACE2 interface (14). Finally, SARS-CoV-2 also has a multi-base (FURIN) cleavage site that can increase the ability of the virus to internalize into cells, thereby reducing its dependence on target cell proteases for entry (15). Based on the abovementioned findings, SARS-CoV-2 exhibits a high affinity with human receptors and notable contagiousness, and its consequences are more serious than other coronaviruses. Intervention strategies based on the SARS-CoV-2 receptor recognition structure are currently studied.

## Clinical Manifestations of COVID-19

### Relationship of ACE2 Distribution and Clinical Manifestations

The clinical manifestations of COVID-19 have a strong correlation with the tissue distribution of ACE2, and its initial clinical manifestations are usually fever, dry cough, shortness of breath, and pneumonia (28). As the prominently targeted organ, ACE2 in normal lung tissue is expressed in type I and type II alveolar epithelial cells (22). The interaction between SARS-CoV2 and ACE2 may cause symptomatic infection. In the second or third week of a symptomatic infection, the infection can develop into a severe disease with dyspnoea and chest symptoms (30). Pathological changes show diffuse alveolar injury with cellular fibromyxoid exudates, pulmonary edema, and hyaline membrane formation, leading to acute respiratory distress syndrome (ARDS) (31). Clinical data show decreased oxygen saturation, and the radiological characteristic is progressive pneumonia (6). Laboratory findings indicate that lymphopenia with or without leukocyte abnormalities is the major para-clinical criterion for patients with COVID-19 infection. SARS-CoV2 can indirectly infect and destroy immune cells (mostly T cells) and macrophages, causing a decrease in lymphocytes, particularly CD8^+^ T cells, and neutrophils may increase, and blood C-reactive protein and erythrocyte sedimentation rate increase (32). Notably, COVID-19 patients have elevated D-dimers. Researches have reported that the prevalence of venous thromboembolism (VTE) in patients with COVID-19 is 25%, and VTE often leads to unfavorable prognosis (25). Severe COVID-19 patients also have elevated levels of pro- and inflammatory cytokines, and cytokine storm may be the primary phenomenon of virus pathogenesis, which can lead to inflammation, lung injury, ARDS, and other organ failures (33). Some patients show involvement of other organs, and some patients presented with cardiovascular system symptoms as their first complaints, such as palpitation and chest distress. In a clinical study involving 41 COVID-19 patients, 12% of patients have developed acute fulminant myocarditis (34). Whether the pathophysiological mechanism of myocardial injury is due to the direct attack on the heart after SARS-CoV-2 interacts with ACE2 still needs further research. The Human Protein Atlas database shows that ACE2 protein has a high expression level in the kidneys (35). A recent study showed that 23 of 85 COVID-19 patients have developed acute renal failure. The autopsy results show that six of the patients have severe acute tubular necrosis (28). Intracellular virus arrays are observed in proximal renal tubular epithelial cells by electron microscopy, indicating that SARS-CoV-2 directly infects human renal tubules and causes acute tubular damage (35). Consequently, deterioration of renal function will increase the burden on the heart and the risk of infection, affecting the poor prognosis. Researchers have explored the expression of ACE2 in the digestive system by scRNA-seq analysis, and the results show that ACE2 is expressed not only in cholangiocytes but also in absorptive enterocytes in the ileum and colon (36). Although gastrointestinal symptoms are not as common as respiratory symptoms, they can also manifest as initial symptoms.

### Potential Transmission Mode of SARS-CoV-2

COVID-19 is primarily spread through respiratory droplets, direct and indirect contact, and has the characteristics of human-to-human transmission. Another mode of transmission is “hidden transmission,” which is defined as asymptomatic virus carriers who become the source of infection and transmit SARS-CoV-2 to close contacts (1). SARS-CoV-2 RNA has also been detected in other biological samples, such as stool, urine, and blood. In particular, stool contains viral RNA in a high percentage of cases, and virus clearance in stool takes longer than pharyngeal swabs. Moreover, the proportion of patients with viral RNA detected in the urine and blood is fairly low (37, 38). Recent studies have reported that SARS-CoV-2 is present in saliva, and the viral load lasts for a long duration; the study has also speculated that the salivary glands may act as a reservoir for SARS-CoV-2 to increase the viral load in saliva (39). Lu et al. reported that SARS-CoV-2 can also be transmitted through the mucous membranes, including conjunctival secretions and tears (40). Therefore, in addition to the respiratory tract and lungs, SARS-CoV-2 transmission raises questions about viral shedding in other body fluids (including seminal fluid) and other modes of transmission. The expression of ACE2 and TMPRSS2 in the testis and male genital tract indicates that the testis is a high-risk organ susceptible to SARS-CoV-2 infection (41). Wang et al. reported that CD147 was another possible SARS-CoV-2 virus invasion pathway. Liu et al. analyzed the expression level of BSG (CD147) and found that BSG was expressed in all types of testis cells (42). The expression of genes involved in multiple pathways provided more possibilities for virus invasion. If the virus can infect human testes, it may involve multiple pathways and even lead to viral contamination of the seminal fluid (42, 43). In previous reports, researchers found that semen samples from survivors of Ebola virus disease remained positive for up to 272 days after the onset of symptoms (44). Some viruses may also be persistent, such as the Zika virus, which can be detected in the semen of a cured male patient for up to 1 year (41). The persistence of the virus indicates that semen can act as a virus reservoir for Ebola and Zika viruses and can be sexually transmitted (9, 10). Is this potential transmission route suitable for SARS-CoV-2? This issue has been under-investigated so far. Examination of existing proteomic databases and sperm surface surveys with monoclonal antibodies revealed that, literally, these cells hold all of the ACEs, including ACE2 (45–48). In addition to ACE2, the fusion between SARS-CoV-2 and human sperm also requires the presence of TMPRSS2. This protease is known to be present in prostasomes that are released into the seminal fluid from the prostate gland at ejaculation (49). These exosome-like structures seem to incorporate TMPRSS2 into sperm (50). A close examination of the human sperm proteomic database also reveals the presence of related proteases TMPRSS11B and TMPRSS12 as well as FURIN (45, 46), in these cells, all of which are thought to serve as activating proteases for viral infection including coronaviruses (51–53). The presence of these activating proteases and ACE2 in the sperm plasma membrane provides the possibility for the sexual transmission of the virus. However, it remains to be seen whether SARS-CoV-2 can replicate in large quantities after entering the cell, and then, release themselves out of host cells causing damage and further spread, just like it does in the lungs. In terms of clinical results, Paoli et al. reported the absence of viral RNA in the semen of a male who was cured by COVID-19 (54). Pan et al. investigated semen samples from 34 Chinese male patients and confirmed the absence of the virus in all samples. Pan et al. also pointed out that in the scRNA-seq dataset of human testicular cells, ACE2, and TMPRSS2 are sparsely expressed in human testes, and there is almost no overlapping gene expression. Therefore, ACE2-mediated viral entry of SARS-CoV-2 into target host cells is unlikely to occur within the human testicle (55). Unfortunately, men with COVID-19 in this study are more likely to have demonstrated milder symptoms. It is plausible that viremia or a certain viral threshold is not achieved to cross the blood-testis barrier (56). Previous studies have shown that higher viral load is associated with more severe disease symptoms. Song et al. tested 12 Chinese patients with COVID-19 at the rehabilitation stage. None of these patients showed viral RNA in their semen samples. Notably, the authors tested the testis tissue of a patient who died of COVID-19 and did not detect viral RNA (57). Contrary to previous results, Li et al. reported the detection of six SARS-CoV-2-positive semen samples in semen collected from 38 severe and recovering Chinese COVID-19 patients (58). The authors cited 12 comatose or dying subjects. As we hypothesized, more severe diseases may correspond to higher blood viral load and a higher chance of crossing the blood-testis barrier. However, the methodological issues of the study have raised some concerns. According to the results of recent clinical studies, SARS-CoV-2 showed only a minor risk of virus shedding into the semen. Nevertheless, even a minor risk is unacceptable in the light of treating otherwise healthy couples for infertility reasons. Therefore, the American Society for Reproductive Medicine and the Society for Assisted Reproductive Technology has issued warning that prospective parents, ART patients, gamete donors, and gestational carriers who meet the SARS-CoV-2 diagnostic criteria must avoid pregnancy or participate in any fertility programs (59). Current studies are limited by the small sample size and short follow-up time. Therefore, detailed information about virus shedding and survival time requires further research. If it could be proved that SARS-CoV-2 can be transmitted sexually in the future studies, the sexual transmission might be a critical part of the prevention of transmission. Based on the abovementioned considerations, patients recovering from SARS-CoV-2 should monitor testicular function, including testosterone and sperm concentration. Unprotected sexual relations must be avoided to prevent from possible infection.

## Impact of Gender on COVID-19 Outcomes

**Figure 2:**
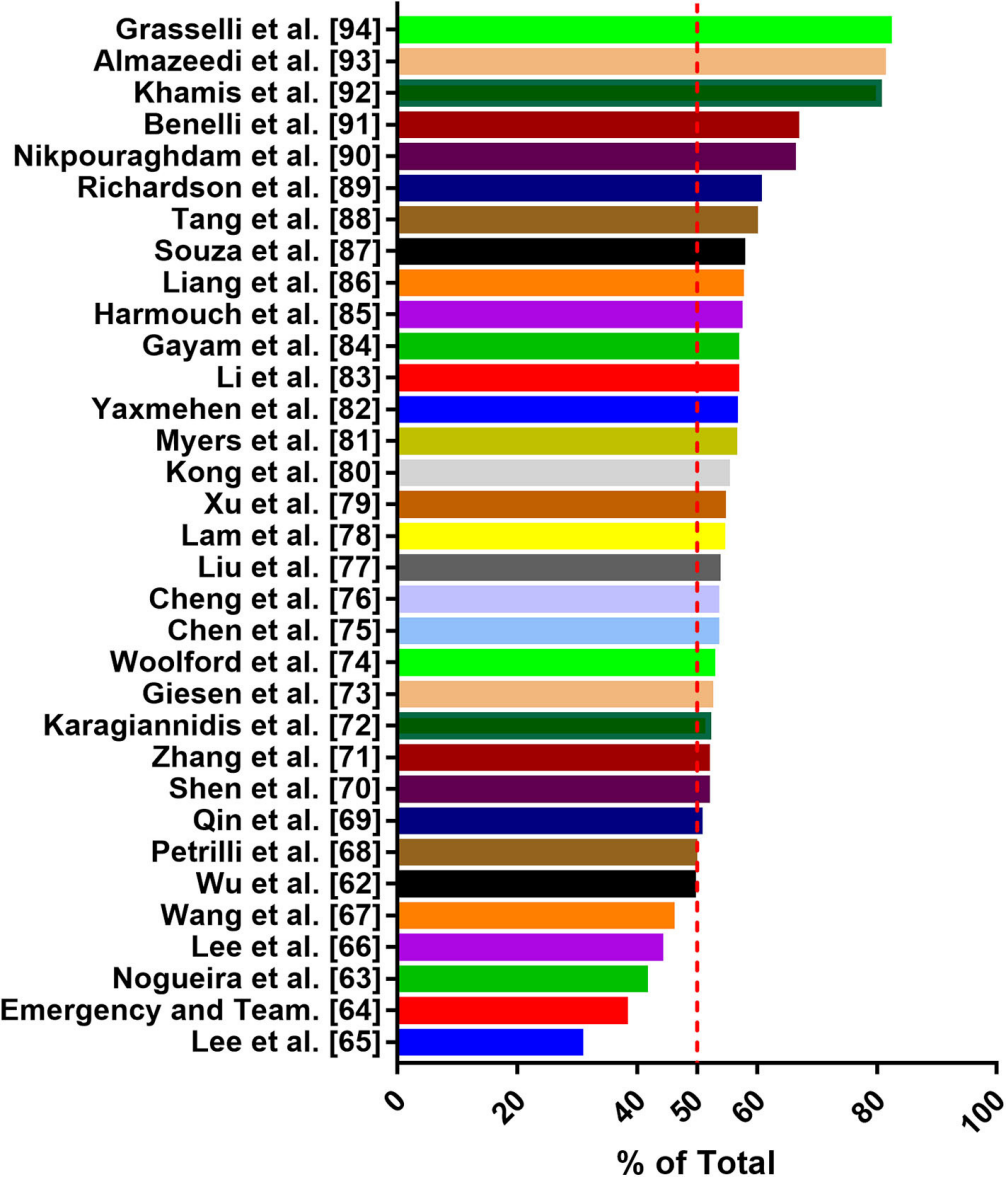
Data on the proportion of males infected with SARS-CoV-2 from 33 articles.

For the first time in China, gender differences in COVID-19-detected cases and mortality has been reported (5, 6). Consistent with the global situation, the difference in the number of cases reported by gender increases progressively in favor of male subjects up to the age group ≥60–69 (66.6%) and ≥70–79 (66.1%). However, in the age group of 20–39, the detection rate of women is slightly high (60). In addition, male individuals are more susceptible to the infection and have the highest mortality rate of SARS-CoV-2 (5). Male represents 73% of deaths in China, 59% in South Korea (25), and 61.8% in the United States (https://www.worldometers.info/coronavirus/coronavirus-age-sexdemographics/). A recent study has collected epidemiological data available to 59,254 patients from 11 different countries, and the results also show an association between male and high mortality rate (61). Several studies have shown that a substantial percentage of COVID-19 occurs in patients with underlying comorbidities. In particular, in elderly male patients with comorbidities, the mortality rate of COVID-19 appears to be higher. Common comorbidities include cardiovascular disease, diabetes, chronic respiratory disease, hypertension, and cancer. Men with pre-existing cardiovascular conditions have the highest case fatality rate (6, 33, 34). Thus, some researchers have considered male sex as a poor prognostic factor (25). Although the research data of Wu et al. (62), Nogueira et al. (63), and Korea Centers for Disease Control and Prevention showed that the proportion of women infected with SARS-CoV-2 was higher, the majority of death cases were men (64). Through literature search and analysis, we found that men are more susceptible to SARS-CoV-2 infection than women (Figure 2) (62–94). Why are men more affected in this pandemic? In this context, analyzing the pathological mechanism of SARS-CoV-2 binding to various tissue cells under different hormone environments is important. Based on previous studies, SARS-Cov-2 interacts with the ACE2 receptor and TMPRSS2 to enter the cell (15). Physiologically, the expression of ACE2 is negatively correlated with age, and men have higher expression than women of comparable age (34). Some reports indicate that healthy and diabetic men and men with renal disease have higher levels of ACE2 circulation than women (95). Sex hormones affect many components of tissue-based RAAS, including ACE2 (96). Although the genes coding for ACE2 are located on the X chromosome, many reports of preclinical studies agree that the expression of ACE2 in males under pathological conditions is frequently higher than that in females (23, 96). Data from experimental animal models has shown that sex hormones can affect the expression and activity of ACE2 in the mouse adipose tissue, kidneys, and myocardium. In normal mice, the activity of ACE2 in the kidneys of male mice is higher than that of female mice; spontaneously hypertensive male mice also show higher ACE2 expression than female mice, and the difference may be due to the secretion of female estradiol (E2) (97, 98). Some studies also show that, after ovariectomy, the ACE2 expression in the kidneys and adipose tissue of women increases, whereas estradiol replacement decreases the expression of ACE2. Male orchiectomy can reduce ACE2 activity. Thus, testosterone maintains high levels of ACE2 expression in the heart and kidneys, whereas estrogen reduces ACE2 expression in these organs (23). Recently, it has been confirmed that the expression level of ACE2 in male lungs is higher than that in females (31). TMPRSS2 belongs to the type II transmembrane serine protease family, which is considered as a critical host cell factor for the spread of a variety of clinically relevant viruses, including influenza A virus, SARS-CoV, and MERS-CoV coronaviruses (3, 99). TMPRSS2 is highly expressed in the prostate epithelium, localized, and metastatic prostate cancer (100). TMPRSS2 expression has also been detected in airway epithelial cells (31). Androgen receptor (AR) activity is considered as a requirement for TMPRSS2 gene transcription (30). AR has been shown to modulate TMPRSS2 expression in non-prostate tissues (including the lungs). *In vitro* and *in vivo* studies have shown that androgen administration increases TMPRSS2 expression in human lung epithelial cells, and androgen deprivation reduces the transcription of TMPRSS2 in the murine lung (101). Moreover, inhibiting TMPRSS2 may prevent SARS-CoV-2 infection (102). Data from a study in the Veneto region of Italy show that among patients with SARS-CoV-2 infection in 68 hospitals, the risk of infection for SARS-CoV-2 in-patients with prostate cancer who received androgen-deprivation therapy (ADT) is significantly reduced (OR 4.05; 95% CI 1.55-10.59) compared with patients who did not receive ADT (100). Collectively, the modulation of testosterone on the expression of ACE2 and TMPRSS2 is considered as a contributor to the dominant male COVID-19 infection (30). However, no thorough analysis of the specific mechanism of this difference has been conducted. In addition to the aforementioned factors, body immune response, viral load, lifestyle differences, and other potentially unknown mechanisms may jointly affect the progress and prognosis of COVID-19. Men and women are known to have differences with regard to the risk and severity of diseases involving the immune system. Women are susceptible to autoimmune diseases, whereas men are disproportionately affected by infectious diseases. In particular, age may lead to gender differentiation in the outcome of COVID-19 cases. Compared with women, men's age-related decline in T cell function accelerates (103). Globally, smoking and drinking rates are higher among men than women, and smoking is associated with increased activity of ACE2. Gender differences in behavior such as smoking and drinking may cause men to have an increased risk of comorbidities, such as chronic lung disease, hypertension, and cardiovascular disease, which may provide a possible explanation for the higher mortality in men (23).

## SARS-CoV-2 and Male Fertility

Similarly, scRNA-seq analysis documents that ACE2 is highly expressed in seminiferous tubule cells, spermatogonia, adult Leydig, and Sertoli cells of the human testis, and Leydig cells may be involved in the regulation of steroidogenesis (104). TMPRSS2 is highly expressed in the prostate epithelial cells and the apical plasma membrane of prostate luminal cells (15). These findings imply a potential risk associated with SARS-CoV-2 infection in the male reproductive system. Based on previous studies, viruses such as HIV, HBV, mumps, human herpes, Ebola, and Zika can invade the human testes and cause viral orchitis, and in some cases, lead to male infertility and testicular tumor (105). Xu et al. described the pathological changes of the testis in the autopsy reports of six men who died of SARS-CoV complications. The testes of the deceased showed extensive germ cell destruction, with few or no sperm in the seminiferous tubules. The basement membrane of the testis was thickened, and peritubular fibrosis was observed. Leukocyte infiltration and vascular congestions were present in the interstitial tissue (8). At present, such occurrence has not yet been described for SARS-CoV-2. A recent study has reported the characteristics of the 34 Chinese men recovering from COVID-19 and found that six patients (19%) have scrotal discomfort around the time of COVID-19 confirmation (55). However, no testicular investigation was conducted in these patients to rule out this aspect and the possibility of viral orchitis remains unclear. We propose hypotheses based on previous studies. Testicular injury in COVID-19 patients may involve multiple possible mechanisms: (1) Fever raises the temperature of the testis, leading to apoptosis of meiotic germ cells (106). Fever also plays an important role in mumps orchitis. (2) Similar to other viral orchitides, SARS-CoV-2 may cross the blood-testis barrier and trigger an immune response in the testis or a secondary autoimmune response, leading to autoimmune orchitis (8, 55). (3) The combination of SARS-CoV-2 and ACE2 may directly impair testicular function and cause epididymal orchitis. (4) COVID-19 has been associated with abnormalities in coagulation, and the segmental vascularization in the testis can account for an orchitis-like syndrome (43). Recently, the world's first case of priapism in a COVID-19 patient was reported, and the presence of dark blood clots at cavernosal blood aspiration supports ischemia-related priapism (107). Previous studies have found that orchitis is a complication of SARS (8). Due to the strict relation between the two viruses, it may be generalized to SARS-CoV-2, but it should be emphasized that the current evidence is limited and contradictory. As mentioned earlier, sperm cells hold all of the ACEs, including ACE2, which converts angiotensin II to angiotensin (1-7). Recent publications indicate that human sperm also express angiotensin II type 1 receptor (AT1R), angiotensin II type 2 receptor (AT2R), and the angiotensin (1-7) MAS receptor (48, 108). These cells, therefore, possess the complete repertoire of ligand-processing enzymes and receptors needed to support RAAS. By analogy with somatic cells, a SARS-CoV-2 attack on human spermatozoa would be expected to impact ACE2 activity leading to an increase in the availability of angiotensin II relative to angiotensin (1-7). Since angiotensin II stimulates the acrosome reaction in sperm cells, it is possible that prolonged exposure to elevated levels of angiotensin II might lead to premature acrosomal exocytosis and sperm senescence (109). Angiotensin II also further affects sperm fertilization and motility by stimulating AT1R and AT2R (108). The recent discovery of MAS receptors in the principal piece of the sperm tail and the acrosomal domain of the sperm head further emphasizes the importance of ACE2. Angiotensin (1-7) activates MAS receptors to maintain sperm in a viable motile state. However, SARS-CoV-2 attack may affect the generation of angiotensin (1-7), thereby initiating a truncated apoptotic cascade characterized by rapid motility loss (110). A cohort study showed that although SARS-CoV-2 RNA was not detected in the semen samples of recovered or acutely infected patients, patients with a moderate infection have statistically significant impairment of sperm quality (sperm concentration, total number of sperm per ejaculate, total number of progressive motility, total number of complete motility) compared with men recovered from a mild infection and the control group (111). The impact of SARS-CoV-2 on male reproductive function is still unclear, and the abovementioned possible pathogenesis needs further research.

Recently, how the COVID-19 pandemic will affect fertility has received widespread attention. Considering the previous pandemic experience and the scale of the COVID-19 pandemic, fertility decline seems to be possible, particularly in high-income countries and in the short term (112). Given the possible impact of SARS-CoV-2 on male fertility, COVID-19 may directly or indirectly affect the world's demographics in the future.

## Conclusion

COVID-19 is a zoonotic coronavirus disease that has constituted a pandemic, endangering human lives, and the global economy (32). Compared with women, men are more susceptible to infections in this outbreak, and their mortality of COVID-19 is also higher (5). SARS-CoV-2 is the etiological agent of COVID-19, which is primarily spread through respiratory droplets, direct and indirect contact (1). Genomic analysis shows that SARS-CoV-2 is 79% identical to the SARS-CoV, and both use ACE2 as their receptor. In addition, TMPRSS2 can enhance ACE2-mediated viral entry (15, 16). The structural basis of SARS-CoV-2 receptor recognition indicates that it has a higher affinity with human ACE2, and the consequences are more serious than other coronaviruses (14). Although ACE2 acts as a “gate” for viruses to invade cells, it also has protective effects on multiple pathophysiological processes (28). The clinical manifestations of COVID-19 have a strong correlation with tissue distribution of ACE2. Apart from the lung tissue, ACE2 is also expressed in the heart, kidney, intestine, and testis and causes corresponding clinical symptoms (21, 22). Testosterone can increase the expression of ACE2 and TMPRSS2, and apart from human immune response, lifestyle differences and other factors affect the progress and prognosis of COVID-19, providing a possible explanation for the male-dominated infection and higher mortality (23, 30, 103). Studies have found that the expression of ACE2 and TMPRSS2 in the testes and male genital tract indicate that the testis is also an organ susceptible to SARS-CoV-2 infection (41). A close examination of the human sperm proteomic database reveals that these cells not only hold all of the ACEs (including ACE2), but also have related proteases TMPRSS11B, TMPRSS12, and FURIN (45–48). The presence of these activating proteases and ACE2 in the sperm plasma membrane provides the possibility for the sexual transmission of the virus. Based on previous studies, many viruses can invade the human testes, cause viral orchitis (8, 105), and even lead to viral contamination of seminal fluid (43). For example, seminal fluid can serve as a virus reservoir for Ebola and Zika viruses, and they can be sexually transmitted (9, 10). But it is still quite unclear for SARS-CoV-2. A study has reported that six Chinese male patients (19%) recovering from COVID-19 have scrotal discomfort around the time of COVID-19 confirmation (55). Li et al. reported the detection of six SARS-CoV-2-positive semen samples in semen collected from 38 severe and recovering Chinese COVID-19 patients (58). However, more studies have reported the opposite result (41, 57). The risk of testicular damage and sexual transmission caused by SARS-CoV-2 infection requires further in-depth studies. How the COVID-19 pandemic will affect fertility has received widespread attention. Recent publications indicate that human sperm also express angiotensin II type 1 receptor (AT1R), angiotensin II type 2 receptor (AT2R), and the angiotensin (1-7) MAS receptor (48, 108). SARS-CoV-2 attacking human sperm may interact with these receptors, affecting sperm fertilization and motility in many ways, leading to male infertility.

This article describes the pathogenic mechanism and clinical manifestations of SARS-CoV-2 infection according to published literature. Based on the epidemiological results, the susceptibility of men to SARS-CoV-2 needs further exploration. The possible mechanism of orchitis caused by SARS-CoV-2 and the potential transmission route of the virus are proposed, raising concerns about male reproductive health in the context of COVID-19.

## Author Contributions

WH and XL searched the literature and conceived and wrote the review. LF, SX, YulL, LC, YuL, GW, DL, and BF critically appraised the literature and made a intellectual contribution to the work. All authors approved the final version of the manuscript for publication.

## Conflict of Interest

The authors declare that the research was conducted in the absence of any commercial or financial relationships that could be construed as a potential conflict of interest.

## References

[1] Al-Qahtani AA Severe acute respiratory syndrome coronavirus 2. (SARS-CoV-2): emergence, history, basic and clinical aspects. Saudi J Biol Sci. (2020) 27:2531–8. 10.1016/j.sjbs.2020.04.033 32336927 PMC7179492

[2] Pal M Berhanu G Desalegn C Kandi V Severe acute respiratory syndrome coronavirus-2 (SARS-CoV-2): an update. Cureus. (2020) 2:e7423 10.7759/cureus.7423 PMC7182166 32337143

[3] De Wit E Van Doremalen N Falzarano D Munster VJ. SARS and MERS: recent insights into emerging coronaviruses. Nat Rev Microbiol. (2016) 14:523–34. 10.1038/nrmicro.2016.81 27344959 PMC7097822

[4] Guan W Ni Z Hu Y Liang W Ou C He J Clinical characteristics of coronavirus disease 2019 in China. N Engl J Med. (2020) 382:1708–20. 10.1056/NEJMoa2002032 32109013 PMC7092819

[5] Li X Xu S Yu M Wang K Tao Y Zhou Y. Risk factors for severity and mortality in adult COVID-19 inpatients in Wuhan. J Allergy Clin Immunol. (2020) 146:110–8. 10.1016/j.jaci.2020.04.006 32294485 PMC7152876

[6] Mo P Xing Y Xiao Y Deng L Zhao Q Wang H. Clinical characteristics of refractory COVID-19 pneumonia in Wuhan, China. Clin Infect Dis. (2020) 16:ciaa270. 10.1093/cid/ciaa270 32173725 PMC7184444

[7] Sigurardóttir ÓG Kolbjornsen O Lutz H. Orchitis in a cat associated with coronavirus infection. J Comp Pathol. (2001) 124:219–22. 10.1053/jcpa.2000.0443 11222021 PMC7130237

[8] Xu J Qi L Chi X Yang J Wei X Gong E. Orchitis: a complication of severe acute respiratory syndrome (SARS). Biol Reprod. (2006) 74:410–6. 10.1095/biolreprod.105.044776 16237152 PMC7109827

[9] Mate SE Kugelman JR Nyenswah TG Ladner JT Wiley MR Cordier-Lassalle T. Molecular evidence of sexual transmission of ebola virus. N Engl J Med. (2015) 373:2448–54. 10.1056/NEJMoa1509773 26465384 PMC4711355

[10] Foy BD Kobylinski KC Foy JLC Blitvich BJ da Rosa AT Haddow AD. Probable non-vector-borne transmission of Zika virus, Colorado, USA. Emerg Infect Dis. (2011) 17:880–2. 10.3201/eid1705.101939 21529401 PMC3321795

[11] Li F. Structure, function, and evolution of coronavirus spike proteins. Ann Rev Virol. (2016) 3:237–61. 10.1146/annurev-virology-110615-042301 27578435 PMC5457962

[12] Walls AC Park YJ Tortorici MA Wall A McGuire AT Veesler D. Structure, function, and antigenicity of the SARS-CoV-2 spike glycoprotein. Cell. (2020) 181:281–92.e6. 10.1016/j.cell.2020.02.058 32155444 PMC7102599

[13] Maier HJ Bickerton E Britton P Coronaviruses: methods and protocols. Coronaviruses Methods Protoc. (2015) 1282:1–282. 10.1007/978-1-4939-2438-7 25870870

[14] Shang J Ye G Shi K Wan Y Luo C Aihara H. Structural basis of receptor recognition by SARS-CoV-2. Nature. (2020) 581:221–4. 10.1038/s41586-020-2179-y 32225175 PMC7328981

[15] Shang J Wan Y Luo C Ye G Geng Q Auerbach A. Cell entry mechanisms of SARS-CoV-2. Proc Natl Acad Sci USA. (2020) 117:11727–34. 10.1073/pnas.2003138117 32376634 PMC7260975

[16] Lu R Zhao X Li J Niu P Yang B Wu H. Genomic characterisation and epidemiology of 2019 novel coronavirus: implications for virus origins and receptor binding. Lancet. (2020) 395:565–74. 10.1016/S0140-6736(20)30251-8 32007145 PMC7159086

[17] Heurich A Hofmann-Winkler H Gierer S Liepold T Jahn O Pohlmann S. TMPRSS2 and ADAM17 cleave ACE2 differentially and only proteolysis by TMPRSS2 augments entry driven by the severe acute respiratory syndrome coronavirus spike protein. J Virol. (2014) 88:1293–307. 10.1128/jvi.02202-13 24227843 PMC3911672

[18] Zhang H Wada J Hida K Tsuchiyama Y Hiragushi K Shikata K. Collectrin, a collecting duct-specific transmembrane glycoprotein, is a novel homolog of ACE2 and is developmentally regulated in embryonic kidneys. J Biol Chem. (2001) 276:17132–9. 10.1074/jbc.M006723200 11278314

[19] Song W Gui M Wang X Xiang Y. Cryo-EM structure of the SARS coronavirus spike glycoprotein in complex with its host cell receptor ACE2. PLoS Pathog. (2018) 14:e1007236. 10.1371/journal.ppat.1007236 30102747 PMC6107290

[20] Li F Li W Farzan M Harrison SC. Structural biology: structure of SARS coronavirus spike receptor-binding domain complexed with receptor. Science. (2005) 309:1864–8. 10.1126/science.1116480 16166518

[21] Donoghue M Hsieh F Baronas E Godbout K Gosselin M Stagliano N. A novel angiotensin-converting enzyme—related to angiotensin 1-9. Circ Res. (2000) 87:e1–9. 10.1161/01.RES.87.5.e1 10969042

[22] Hamming I Timens W Bulthuis MLC Lely AT Navis GJ van Goor H. Tissue distribution of ACE2 protein, the functional receptor for SARS coronavirus. A first step in understanding SARS pathogenesis. J Pathol. (2004) 203:631–7. 10.1002/path.1570 15141377 PMC7167720

[23] Klein SL Morgan R. The impact of sex and gender on immunotherapy outcomes. Biol Sex Differ. (2020) 11:24. 10.1186/s13293-020-00301-y 32366281 PMC7197158

[24] Review I. Immune activation and inflammation in HIV-1 infection: causes and consequences. J Pathol. (2008) 214:231–41. 10.1002/path 18161758

[25] La Vignera S Cannarella R Condorelli RA Torre F Aversa A Calogero AE. Sex-specific SARS-CoV2 mortality: among hormone-modulated ace2 expression, risk of venous thromboembolism and hypovitaminosis D. Int J Mol Sci. (2020) 21:5–10. 10.3390/ijms21082948 32331343 PMC7215653

[26] Huang F Guo J Zou Z Liu J Cao B Zhang S. Angiotensin II plasma levels are linked to disease severity and predict fatal outcomes in H7N9-infected patients. Nat Commun. (2014) 5:3595. 10.1038/ncomms4595 24800963 PMC7091598

[27] Zou Z Yan Y Shu Y Gao R Sun Y Li X. Angiotensin-converting enzyme 2 protects from lethal avian influenza A H5N1 infections. Nat Commun. (2014) 5:3594. 10.1038/ncomms4594 24800825 PMC7091848

[28] Yan T Xiao R Lin G. Angiotensin-converting enzyme 2 in severe acute respiratory syndrome coronavirus and SARS-CoV-2: a double-edged sword? FASEB J. (2020) 34:6017–26. 10.1096/fj.202000782 32306452 PMC7264803

[29] Chen Y Guo Y Pan Y Zhao ZJ. Structure analysis of the receptor binding of 2019-nCoV. Biochem Biophys Res Commun. (2020) 525:135–40. 10.1016/j.bbrc.2020.02.071 32081428 PMC7092824

[30] Pozzilli P Lenzi A. Testosterone, a key hormone in the context of COVID-19 pandemic. Metabolism. (2020) 108:154252. 10.1016/j.metabol.2020.154252 32353355 PMC7185012

[31] Zhao Y Zhao Z Wang Y Zhou Y Ma Y Zuo W. Single-cell RNA expression profiling of ACE2, the receptor of SARS-CoV-2. Am J Respir Crit Care Med. (2020) 202:756–9. 10.1164/rccm.202001-0179LE 32663409 PMC7462411

[32] Tahmasebi S Khosh E Esmaeilzadeh A. The outlook for diagnostic purposes of the 2019-novel coronavirus disease. J Cell Physiol. (2020) 235:9211–29. 10.1002/jcp.29804 32452050 PMC7283732

[33] Huang C Wang Y Li X Ren L Zhao J Hu Y. Clinical features of patients infected with 2019 novel coronavirus in Wuhan, China. Lancet. (2020) 395:497–506. 10.1016/S0140-6736(20)30183-5 31986264 PMC7159299

[34] Zheng YY Ma YT Zhang JY Xie X. COVID-19 and the cardiovascular system. Nat Rev Cardiol. (2020) 17:259–60. 10.1038/s41569-020-0360-5 32139904 PMC7095524

[35] Chen L Guo C. Focus on kidney disease among the coronavirus disease 2019 patients: a comparative perspective between China, Italy and the United States. Int J Clin Pract. (2020) 74:e13561. 10.1111/ijcp.13561 32460395 PMC7283811

[36] Zhang H Kang Z Gong H Xu D Wang J Li Z The digestive system is a potential route of 2019-nCov infection: a bioinformatics analysis based on single-cell transcriptomes. bioRxiv [Preprint]. (2020). 10.1101/2020.01.30.927806

[37] Ling Y Xu SB Lin YX Tian D Zhu ZQ Dai FH. Persistence and clearance of viral RNA in 2019 novel coronavirus disease rehabilitation patients. Chin Med J. (2020) 133:1039–43. 10.1097/CM9.0000000000000774 32118639 PMC7147278

[38] Xie C Jiang L Huang G Pu H Gong B Lin H. Comparison of different samples for 2019 novel coronavirus detection by nucleic acid amplification tests. Int J Infect Dis. (2020) 93:264–7. 10.1016/j.ijid.2020.02.050 32114193 PMC7129110

[39] Song J Li Y Huang X Chen Z Li Y Liu C Systematic analysis of ACE2 and TMPRSS2 expression in salivary glands reveals underlying transmission mechanism caused by SARS-CoV-2. J Med Virol. (2020) 92:2556–66. 10.1002/jmv.26045 PMC7280739 32441816

[40] Xia J Tong J Liu M Shen Y Guo D. Evaluation of coronavirus in tears and conjunctival secretions of patients with SARS-CoV-2 infection. J Med Virol. (2020) 92:589–94. 10.1002/jmv.25725 32100876 PMC7228294

[41] Paoli D Pallotti F Turriziani O Mazzuti L Antonelli G Lenzi A SARS-CoV-2 presence in seminal fluid: myth or reality. Andrology. (2020) 1–4. 10.1111/andr.12825 32453494 PMC7283802

[42] Liu X Chen Y Tang W Zhang L Chen W Yan Z. Single-cell transcriptome analysis of the novel coronavirus (SARS-CoV-2) associated gene ACE2 expression in normal and non-obstructive azoospermia (NOA) human male testes. Sci China Life Sci. (2020) 63:1006–15. 10.1007/s11427-020-1705-0 32361911 PMC7195615

[43] Corona G Baldi E Isidori AM Paoli D Pallotti F De Santis L. SARS-CoV-2 infection, male fertility and sperm cryopreservation: a position statement of the Italian society of andrology and sexual medicine (SIAMS) (Società Italiana di Andrologia e Medicina della Sessualità). J Endocrinol Invest. (2020) 43:1153–7. 10.1007/s40618-020-01290-w 32462316 PMC7252417

[44] Deen GF Broutet N Xu W Knust B Sesay FR McDonald SLR. Ebola RNA persistence in semen of ebola virus disease survivors — final report. N Engl J Med. (2017) 377:1428–37. 10.1056/NEJMoa1511410 26465681 PMC5798881

[45] Baker MA Reeves G Hetherington L Müller J Baur I Aitken RJ. Identification of gene products present in triton X-100 soluble and insoluble fractions of human spermatozoa lysates using LC-MS/MS analysis. Proteomics Clin Appl. (2007) 1:524–32. 10.1002/prca.200601013 21136703

[46] Castillo J Jodar M Oliva R. The contribution of human sperm proteins to the development and epigenome of the preimplantation embryo. Hum Reprod Update. (2018) 24:535–55. 10.1093/humupd/dmy017 29800303

[47] Wang Y Wan J Ling X Liu M Zhou T. Front cover: the human sperm proteome 2.0: an integrated resource for studying sperm functions at the level of post-translational modification. Proteomics. (2016) 16:2597–601. 10.1002/pmic.201670170 27546384

[48] Valdivia A Cortés L Beitia M Totorikaguena L Agirregoitia N Corcostegui B. Role of angiotensin-(1-7) via MAS receptor in human sperm motility and acrosome reaction. Reproduction. (2020) 159:241–9. 10.1530/REP-19-0274 31869308

[49] Chen YW Lee MS Lucht A Chou FP Huang W Havighurst TC. TMPRSS2, a serine protease expressed in the prostate on the apical surface of luminal epithelial cells and released into semen in prostasomes, is misregulated in prostate cancer cells. Am J Pathol. (2010) 176:2986–96. 10.2353/ajpath.2010.090665 20382709 PMC2877858

[50] Saez F Sullivan R. Prostasomes, post-testicular sperm maturation and fertility. Front Biosci Landmark. (2016) 21:1464–73. 10.2741/4466 27100516

[51] Yun B Zhang Y Liu Y Guan X Wang Y Qi X. TMPRSS12 is an activating protease for subtype B avian metapneumovirus. J Virol. (2016) 90:11231–46. 10.1128/jvi.01567-16 27707927 PMC5126379

[52] Ji HL Zhao R Matalon S Matthay MA. Elevated plasmin(ogen) as a common risk factor for COVID-19 susceptibility. Physiol Rev. (2020) 100:1065–75. 10.1152/physrev.00013.2020 32216698 PMC7191627

[53] Singh M Bansal V Feschotte C. A single-cell RNA expression map of human coronavirus entry factors. SSRN. (2020) 32:108175. 10.1016/j.celrep.2020.108175 32946807 PMC7470764

[54] Paoli D Pallotti F Colangelo S Basilico F Mazzuti L Turriziani O. Study of SARS-CoV-2 in semen and urine samples of a volunteer with positive naso-pharyngeal swab. J Endocrinol Invest. (2020) 23:1–4. 10.1007/s40618-020-01261-1 32329026 PMC7179792

[55] Pan F Xiao X Guo J Song Y Li H Patel DP. No evidence of severe acute respiratory syndrome–coronavirus 2 in semen of males recovering from coronavirus disease 2019. Fertil Steril. (2020) 113:1135–9. 10.1016/j.fertnstert.2020.04.024 32482249 PMC7164916

[56] Liu Y Yan LM Wan L Xiang TX Le A Liu JM. Viral dynamics in mild and severe cases of COVID-19. Lancet Infect Dis. (2020) 20:656–7. 10.1016/S1473-3099(20)30232-2 32199493 PMC7158902

[57] Song C Wang Y Li W Hu B Chen G Xia P. Absence of 2019 novel coronavirus in semen and testes of COVID-19 patients. Biol Reprod. (2020) 103:4–6. 10.1093/biolre/ioaa050 32297920 PMC7184456

[58] Li D Jin M Bao P Zhao W Zhang S. Clinical characteristics and results of semen tests among men with coronavirus disease 2019. JAMA Netw Open. (2020) 3:e208292. 10.1001/jamanetworkopen.2020.8292 32379329 PMC7206502

[59] Cardona Maya WD Du Plessis SS Velilla PA. SARS-CoV-2 and the testis: similarity with other viruses and routes of infection. Reprod Biomed Online. (2020) 40:763–4. 10.1016/j.rbmo.2020.04.009 32362571 PMC7162782

[60] Walter LA McGregor AJ. Sex- and gender-specific observations and implications for COVID-19. West J Emerg Med. (2020) 21:507–9. 10.5811/westjem.2020.4.47536 32302282 PMC7234726

[61] Borges do Nascimento IJ Cacic N Abdulazeem HM von Groote TC Jayarajah U Weerasekara I. Novel coronavirus infection (COVID-19) in humans: a scoping review and meta-analysis. J Clin Med. (2020) 9:941. 10.3390/jcm9040941 32235486 PMC7230636

[62] Wu Y Guo W Liu H Qi B Liang K Xu H. Clinical outcomes of 402 patients with COVID-2019 from a single center in Wuhan, China. J Med Virol. (2020) 92:2751–7. 10.1002/jmv.26168 32530494 PMC7307018

[63] Nogueira PJ de Araújo Nobre M Costa A Ribeiro RM Furtado C Bacelar Nicolau L The role of health preconditions on COVID-19 deaths in portugal: evidence from surveillance data of the first 20293 infection cases. J Clin Med. (2020) 9:2368 10.3390/jcm9082368 PMC7464004 32722159

[64] Emergency CN Team CM Osong public health and research perspectives. Osong Public Heal Res Perspect. (2012) 3:62 10.1016/j.phrp.2012.03.003

[65] Yeon LJ Hong SW Hyun M Park JS Lee JH Suh YS. Epidemiological and clinical characteristics of coronavirus disease 2019 in Daegu, South Korea. Int J Infect Dis. (2020) 98:462–6. 10.1016/j.ijid.2020.07.017 32702415 PMC7371586

[66] Lee SW Ha EK Yeniova AÖ Moon SY Kim SY Koh HY. Severe clinical outcomes of COVID-19 associated with proton pump inhibitors: a nationwide cohort study with propensity score matching. Gut. (2020) 1–9. 10.1136/gutjnl-2020-322248 32732368

[67] Wang X Pan Y Zhang D Chen L Jia L Li X. Basic epidemiological parameter values from data of real-world in mega-cities: the characteristics of COVID-19 in Beijing, China. BMC Infect Dis. (2020) 20:526. 10.1186/s12879-020-05251-9 32689956 PMC7370267

[68] Petrilli CM Jones SA Yang J Rajagopalan H O'Donnell L Chernyak Y. Factors associated with hospital admission and critical illness among 5279 people with coronavirus disease 2019 in New York City: Prospective cohort study. BMJ. (2020) 369:m1966. 10.1136/bmj.m1966 32444366 PMC7243801

[69] Qin C Zhou L Hu Z Yang S Zhang S Chen M. Clinical characteristics and outcomes of COVID-19 patients with a history of stroke in Wuhan, China. Stroke. (2020) 2219–23. 10.1161/STROKEAHA.120.030365 32466735 PMC7282412

[70] Shen Y Zheng F Sun D Ling Y Chen J Li F. Epidemiology and clinical course of COVID-19 in Shanghai, China. Emerg Microbes Infect. (2020) 9:1537–45. 10.1080/22221751.2020.1787103 32573353 PMC7473125

[71] Zhang SY Lian JS Hu JH Zhang XL Lu YF Cai H. Clinical characteristics of different subtypes and risk factors for the severity of illness in patients with COVID-19 in Zhejiang, China. Infect Dis Poverty. (2020) 9:85. 10.1186/s40249-020-00710-6 32641121 PMC7341711

[72] Karagiannidis C Mostert C Hentschker C Voshaar T Malzahn J Schillinger G. Case characteristics, resource use, and outcomes of 10 021 patients with COVID-19 admitted to 920 German hospitals: an observational study. Lancet Respir Med. (2020) 8:853–62. 10.1016/S2213-2600(20)30316-7 32735842 PMC7386882

[73] Giesen C Diez-Izquierdo L Saa-Requejo CM Lopez-Carrillo I Lopez-Vilela CA Seco-Martinez A. Epidemiological characteristics of the COVID-19 outbreak in a secondary hospital in Spain. Am J Infect Control. (2020) 70:232–6. 10.1016/j.ajic.2020.07.014 32663494 PMC7834034

[74] Woolford SJ D'Angelo S Curtis EM Parsons CM Ward KA Dennison EM. COVID-19 and associations with frailty and multimorbidity: a prospective analysis of UK Biobank participants. Aging Clin Exp Res. (2020) 32:1897–905. 10.1007/s40520-020-01653-6 32705587 PMC7377312

[75] Chen FF Zhong M Liu Y Zhang Y Zhang K Su D. characteristics and outcomes of 681 severe cases with COVID-19 in China. J Crit Care. (2020) 60:32–7. 10.1016/j.jcrc.2020.07.003 32736197 PMC7340593

[76] Cheng JL Huang C Zhang GJ Liu DW Li P Lu CY. [Epidemiological characteristics of novel coronavirus pneumonia in Henan]. Zhonghua Jie He He Hu Xi Za Zhi. (2020) 43:327–31. 10.3760/cma.j.cn112147-20200222-00148 32118390

[77] Liu J Zhang S Wu Z Shang Y Dong X Li G Clinical outcomes of COVID-19 in Wuhan, China: a large cohort study. Ann Intensive Care. (2020) 10:99 10.1186/s13613-020-00706-3 32737627 PMC7393341

[78] Lam HY Lam TS Wong CH Lam WH Leung CME Au KWA. The epidemiology of COVID-19 cases and the successful containment strategy in Hong Kong–January to May 2020. Int J Infect Dis. (2020) 98:51–8. 10.1016/j.ijid.2020.06.057 32579906 PMC7306206

[79] Xu PP Tian RH Luo S Zu ZY Fan B Wang XM. Risk factors for adverse clinical outcomes with COVID-19 in China: A multicenter, retrospective, observational study. Theranostics. (2020) 10:6372–83. 10.7150/thno.46833 32483458 PMC7255028

[80] Kong W Wang Y Hu J Chughtai A Pu H. Comparison of clinical and epidemiological characteristics of asymptomatic and symptomatic SARS-CoV-2 infection: a multi-center study in Sichuan Province, China. Travel Med Infect Dis. (2020) 37:101754. 10.1016/j.tmaid.2020.101754 32492485 PMC7833588

[81] Myers LC Parodi SM Escobar GJ Liu VX. Characteristics of hospitalized adults with COVID-19 in an integrated health care system in California. JAMA. (2020) 323:2195–8. 10.1001/jama.2020.7202 32329797 PMC7182961

[82] Yaxmehen BCO Armando GD Eduardo AVN Alejandro MS Arsenio VV Paola BLJ Unequal impact of structural health determinants and comorbidity on COVID-19 severity and lethality in older mexican adults: considerations beyond chronological aging. J Gerontol A Biol Sci Med Sci. 29:glaa163. 10.1093/gerona/glaa163 PMC7337730 32598450

[83] Li Q Guan X Wu P Wang X Zhou L Tong Y. Early transmission dynamics in Wuhan, China, of novel coronavirus-infected pneumonia. N Engl J Med. (2020) 382:1199–207. 10.1056/NEJMoa2001316 31995857 PMC7121484

[84] Gayam V Chobufo MD Merghani MA Lamichanne S Garlapati PR Quist J. Clinical characteristics and predictors of mortality in African Americans with COVID-19 from an inner-city community hospital in New York. SSRN Electron J. (2020) 1–8. 10.2139/ssrn.3605246 32672844 PMC7405133

[85] Harmouch F Shah K Hippen J Kumar A Goel H. Is it all in the heart? Myocardial injury as major predictor of mortality among hospitalized COVID-19 patients. J Med Virol. (2020) 1–10. 10.1002/jmv.26347 32710646

[86] Liang WH Guan WJ Li CC Li YM Liang HR Zhao Y. Clinical characteristics and outcomes of hospitalised patients with COVID-19 treated in Hubei (epicentre) and outside Hubei (non-epicentre): a nationwide analysis of China. Eur Respir J. (2020) 55:2000562. 10.1183/13993003.00562-2020 32269086 PMC7144336

[87] Souza WM Fletcher Buss L Candido DS Carrera JP Li S Zarebski AE Epidemiological and clinical characteristics of the early phase of the COVID-19 epidemic in Brazil. medRxiv [Preprint]. (2020) 19 10.1101/2020.04.25.20077396 32737472

[88] Tang N Bai H Chen X Gong J Li D Sun Z Anticoagulant treatment is associated with decreased mortality in severe coronavirus disease 2019 patients with coagulopathy. J Thromb Haemost. (2020) 18:1094–9. 10.1111/jth.14817 32220112 PMC9906401

[89] Richardson S Hirsch JS Narasimhan M Crawford JM McGinn T Davidson KW. Presenting characteristics, comorbidities, and outcomes among 5700 patients hospitalized with COVID-19 in the New York City Area. JAMA. (2020) 323:2052–9. 10.1001/jama.2020.6775 32320003 PMC7177629

[90] Nikpouraghdam M Jalali Farahani A Alishiri GH Heydari S Ebrahimnia M Samadinia H. Epidemiological characteristics of coronavirus disease 2019 (COVID-19) patients in IRAN: A single center study. J Clin Virol. (2020) 127:104378. 10.1016/j.jcv.2020.104378 32353762 PMC7172806

[91] Benelli G Buscarini E Canetta C La Piana G Merli G Scartabellati A SARS-COV-2 comorbidity network and outcome in hospitalized patients in Crema, Italy. medRxiv [Preprint]. (2020). 10.1101/2020.04.14.20053090 PMC7993836 33765013

[92] Khamis F Al Rashidi B Al-Zakwani I Al Wahaibi AH Al Awaidy ST. Epidemiology of COVID-19 infection in Oman: analysis of the first 1304 cases. Oman Med J. (2020) 35:e145. 10.5001/omj.2020.60 32647593 PMC7335452

[93] Almazeedi S Al-Youha S Jamal MH Al-Haddad M Al-Muhaini A Al-Ghimlas F. Characteristics, risk factors and outcomes among the first consecutive 1096 patients diagnosed with COVID-19 in Kuwait. EClinicalMedicine. (2020) 24:100448. 10.1016/j.eclinm.2020.100448 32766546 PMC7335246

[94] Grasselli G Zangrillo A Zanella A Antonelli M Cabrini L Castelli A. Baseline characteristics and outcomes of 1591 patients infected with SARS-CoV-2 admitted to icus of the lombardy region, Italy. JAMA. (2020) 323:1574–81. 10.1001/jama.2020.5394 32250385 PMC7136855

[95] Patel SK Velkoska E Burrell LM. Emerging markers in cardiovascular disease: Where does angiotensin-converting enzyme 2 fit in? Clin Exp Pharmacol Physiol. (2013) 40:551–9. 10.1111/1440-1681.12069 23432153

[96] Chappell MC Marshall AC Alzayadneh EM Shaltout HA Diz DI. Update on the angiotensin converting enzyme 2-angiotensin (1-7)-mas receptor axis: fetal programing, sex differences, and intracellular pathways. Front Endocrinol. (2014) 5:201. 10.3389/fendo.2013.00201 24409169 PMC3886117

[97] Dalpiaz PLM Lamas AZ Caliman IF Ribeiro RF Abreu GR Moyses MR Sex hormones promote opposite effects on ACE and ACE2 activity, hypertrophy and cardiac contractility in spontaneously hypertensive rats. PLoS ONE. (2015) 10:e0127515 10.1371/journal.pone.0127515 26010093 PMC4444272

[98] Ji H Menini S Zheng W Pesce C Wu X Sandberg K. Role of angiotensin-converting enzyme 2 and angiotensin(1-7) in 17β-oestradiol regulation of renal pathology in renal wrap hypertension in rats. Exp Physiol. (2008) 93:648–57. 10.1113/expphysiol.2007.041392 18296494

[99] Shen LW Mao HJ Wu YL Tanaka Y Zhang W. TMPRSS2: a potential target for treatment of influenza virus and coronavirus infections. Biochimie. (2017) 142:1–10. 10.1016/j.biochi.2017.07.016 28778717 PMC7116903

[100] Montopoli M Zumerle S Vettor R Rugge M Zorzi M Catapano CV. Androgen-deprivation therapies for prostate cancer and risk of infection by SARS-CoV-2: a population-based study (N = 4532). Ann Oncol. (2020) 31:1040–5. 10.1016/j.annonc.2020.04.479 32387456 PMC7202813

[101] Mikkonen L Pihlajamaa P Sahu B Zhang FP Jänne OA. Androgen receptor and androgen-dependent gene expression in lung. Mol Cell Endocrinol. (2010) 317:14–24. 10.1016/j.mce.2009.12.022 20035825

[102] Hoffmann M Kleine-Weber H Schroeder S Krüger N Herrler T Erichsen S. SARS-CoV-2 cell entry depends on ACE2 and TMPRSS2 and is blocked by a clinically proven protease inhibitor. Cell. (2020) 181:271–80.e8. 10.1016/j.cell.2020.02.052 32142651 PMC7102627

[103] Márquez EJ Trowbridge J Kuchel GA Banchereau J Ucar D. The lethal sex gap: COVID-19. Immun Ageing. (2020) 17:13. 10.1186/s12979-020-00183-z 32457811 PMC7240166

[104] Wang Z Xu X. scRNA-seq profiling of human testes reveals the presence of the ACE2 receptor, a target for SARS-CoV-2 infection in spermatogonia, leydig and sertoli cells. Cells. (2020) 9:920. 10.3390/cells9040920 32283711 PMC7226809

[105] Koukila-Kahkola P Paulin L Brander E Jantzen E Eho-Remes M Katila ML. Characterisation of a new isolate of *Mycobacterium shimoidei* from Finland. J Med Microbiol. (2000) 49:937–40. 10.1099/0022-1317-49-10-937 11023191

[106] Xu J Xu Z Jiang Y Qian X Huang Y. Cryptorchidism induces mouse testicular germ cell apoptosis and changes in bcl-2 and bax protein expression. J Environ Pathol Toxicol Oncol. (2000) 19:25–33. 10905505

[107] Lamamri M Chebbi A Mamane J Abbad S Munuzzolini M Sarfati F. Priapism in a patient with coronavirus disease 2019. (COVID-19): a case report. Am J Emerg Med. (2020) S0735-6757(20)30514-3. 10.1016/j.ajem.2020.06.027 32732087 PMC7301054

[108] Aitken RJ. COVID-19 and human spermatozoa—potential risks for infertility and sexual transmission? Andrology. (2020) 19. 10.1111/andr.12859 32649023 PMC7404878

[109] Köhn FM Müller G Drescher D Neukamm G El Mulla KF Henkel R. Effect of angiotensin converting enzyme (ACE) and angiotensins on human sperm functions. Andrologia. (1998) 30:207–15. 10.1111/j.1439-0272.1998.tb01162.x 9739417

[110] Koppers AJ Mitchell LA Wang P Lin M Aitken RJ. Phosphoinositide 3-kinase signalling pathway involvement in a truncated apoptotic cascade associated with motility loss and oxidative DNA damage in human spermatozoa. Biochem J. (2011) 436:687–98. 10.1042/BJ20110114 21470189

[111] Holtmann N Edimiris P Andree M Doehmen C Baston-Buest D Adams O. Assessment of SARS-CoV-2 in human semen—a cohort study. Fertil Steril. (2020) 114:233–8. 10.1016/j.fertnstert.2020.05.028 32650948 PMC7256599

[112] Aassve A Cavalli N Mencarini L Plach S Livi Bacci M. The COVID-19 pandemic and human fertility. Science. (2020) 369:370–1. 10.1126/science.abc9520 32703862

